# Neonatal and adult cardiac fibroblasts exhibit inherent differences in cardiac regenerative capacity

**DOI:** 10.1016/j.jbc.2023.104694

**Published:** 2023-04-10

**Authors:** Hualing Sun, Richard E. Pratt, Victor J. Dzau, Conrad P. Hodgkinson

**Affiliations:** 1 Mandel Center for Heart and Vascular Research, and the Duke Cardiovascular Research Center, Duke University Medical Center, Durham, North Carolina, USA; 2 Department of Periodontology, School and Hospital of Stomatology, Wuhan University, Hubei Province, Wuhan, China

**Keywords:** fibroblast, reprogramming, aging, myocardial infarction, microRNA (miRNA)

## Abstract

Directly reprogramming fibroblasts into cardiomyocytes improves cardiac function in the infarcted heart. However, the low efficacy of this approach hinders clinical applications. Unlike the adult mammalian heart, the neonatal heart has an intrinsic regenerative capacity. Consequently, we hypothesized that birth imposes fundamental changes in cardiac fibroblasts which limit their regenerative capabilities. In support, we found that reprogramming efficacy *in vitro* was markedly lower with fibroblasts derived from adult mice *versus* those derived from neonatal mice. Notably, fibroblasts derived from adult mice expressed significantly higher levels of pro-angiogenic genes. Moreover, under conditions that promote angiogenesis, only fibroblasts derived from adult mice differentiated into tube-like structures. Targeted knockdown screening studies suggested a possible role for the transcription factor Epas1. Epas1 expression was higher in fibroblasts derived from adult mice, and Epas1 knockdown improved reprogramming efficacy in cultured adult cardiac fibroblasts. Promoter activity assays indicated that Epas1 functions as both a transcription repressor and an activator, inhibiting cardiomyocyte genes while activating angiogenic genes. Finally, the addition of an Epas1 targeting siRNA to the reprogramming cocktail markedly improved reprogramming efficacy *in vivo* with both the number of reprogramming events and cardiac function being markedly improved. Collectively, our results highlight differences between neonatal and adult cardiac fibroblasts and the dual transcriptional activities of Epas1 related to reprogramming efficacy.

Cardiac injury results in the irreversible and permanent loss of cardiomyocytes (1). Replenishing the cardiomyocyte population still eludes medical science. We were the first to demonstrate that fibroblasts within cardiac scar tissue could be reprogrammed into cardiomyocytes *via* a set of four miRNAs (miR-1, miR-133, miR-208, miR-499) which we called miR combo (2, 3, 4, 5, 6, 7, 8, 9, 10). While we use miRNAs, other groups take advantage of specific combinations of transcription factors (11, 12). Irrespective of whether miRNAs or transcription factors are employed, functional improvements in the infarcted heart are relatively modest (3, 12, 13). The field has sought to improve efficacy by elucidating and then applying mechanistic insights (5, 6, 14, 15, 16, 17). These mechanistic studies have identified important roles for various epigenetic events and innate immunity (5, 6, 14, 15, 16, 17). Mechanistic studies have solely utilized cardiac fibroblasts derived from neonatal mice as these cells are significantly easier to reprogram when compared to adult cardiac fibroblasts (5, 6, 14, 15, 16, 17). Birth appears to induce fibroblasts to undergo significant phenotypic changes (18, 19, 20). Neonatal cardiac fibroblasts produce larger amounts of collagen and lower amounts of cytokines than their adult counterparts (19, 21). Moreover, neonatal cardiac fibroblasts are highly proliferative while adult cardiac fibroblasts are quiescent (19, 21). We hypothesized that the therapeutic benefits of reprogramming could be improved by identifying and manipulating the mechanism whereby fibroblasts transition from their neonatal to adult phenotype.

In this study, we wanted to identify the changes in fibroblasts after birth and how these changes could be manipulated for therapeutic benefits. We found that neonatal and adult cardiac fibroblasts had distinct phenotypes. While neonatal cardiac fibroblasts displayed a pro-cardiomyogenic phenotype, adult cardiac fibroblasts displayed a pro-angiogenic phenotype. The transition from the neonatal to adult cardiac fibroblast phenotype was found to be dependent on the transcription factor Epas1. Promoter activity assays showed that Epas1 played a dual role: stimulating angiogenic genes while inhibiting cardiomyocyte genes. Reverting adult cardiac fibroblasts to their neonatal phenotype *via* Epas1 knockdown improved reprogramming efficacy in adult mice. Importantly, improved reprogramming efficacy *in vivo* also enhanced the functional improvements associated with reprogramming in infarcted adult mice.

## Results

**Figure 1:**
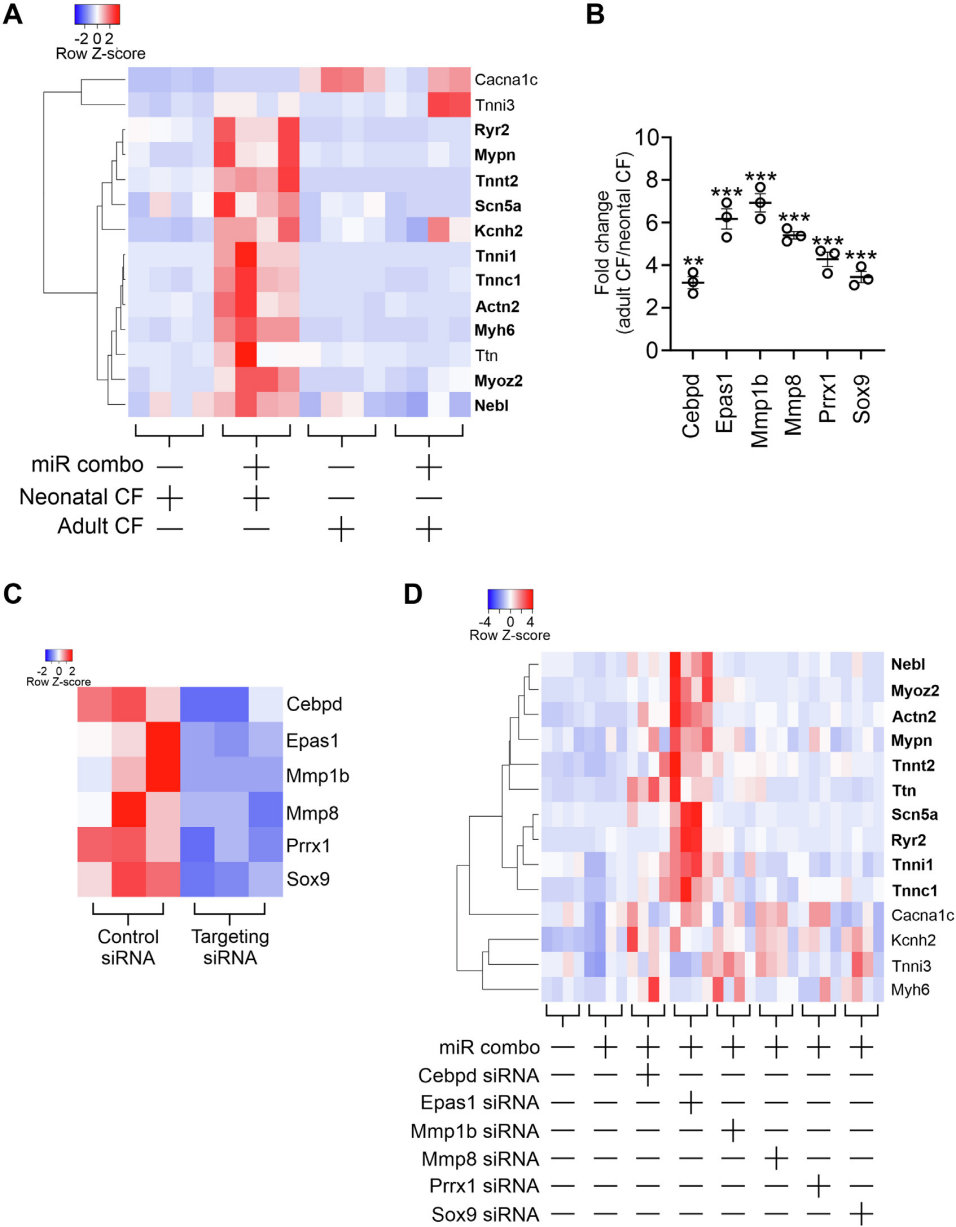
**Reduced fibroblast to cardiomyocyte reprogramming efficacy in adult cardiac fibroblasts.***A*, fibroblasts were isolated from neonatal and adult cardiac tissue. After one passage, cells were transfected with either the non-targeting miRNA negmiR or the reprogramming miRNA cocktail miR combo. After 14 days, expression of the indicated cardiomyocyte-specific markers was determined by qPCR. Expression values were normalized to the housekeeping gene Gapdh. The heatmap shows the expression values (as Z-scores) of four independent experiments. Raw expression data can be found in Table S1. To determine significance, one-way ANOVA was employed on the raw expression data with Bonferroni *post hoc* tests to determine significance between groups. Results from one-way ANOVA and Bonferroni *post hoc* tests are reported in Table S2. Genes for which significant reprogramming (*p* < 0.05) was observed in neonatal cardiac fibroblasts are shown in bold. *B*, the expression of the indicated genes in adult and neonatal cardiac fibroblasts (passage 1) was determined by qPCR. Data are represented as a fold change in expression between adult *versus* neonatal cardiac fibroblasts. N = 3. To determine significance, one-way ANOVA was employed, *F* (6, 14) = 40.91 *p* < 0.0001, with Bonferroni *post hoc* tests to determine significances between groups (∗∗*p* < 0.01, ∗∗∗*p* < 0.001). *C*, adult cardiac fibroblasts were transfected with a control siRNA or a siRNA targeting Cebpd, Epas1, Mmp1, Mmp8, Prrx1, or Sox9. After 3 days, cells were analyzed for the expression of the indicated genes. Expression values were normalized to the housekeeping gene Gapdh. The heatmap shows the expression values (as Z-scores) of three independent experiments. Raw expression data can be found in Table S1. To determine significance, two-way independent T-tests were conducted. All genes were successfully targeted (*p* < 0.05) by their respective siRNAs. *D*, fibroblasts were isolated from adult cardiac tissue. After one passage, cells were transfected with the indicated combination of miRNAs (negmiR or miR combo) and siRNAs (non-targeting control or a siRNA targeting one of the following: Prx1, Cebpd, Epas1, Sox9, Mmp1b, or Mmp8). After 14 days, expression of the indicated cardiomyocyte-specific markers was determined by qPCR. Expression values were normalized to the housekeeping gene Gapdh. The heatmap shows the individual expression values (as Z-scores) of four independent experiments. Raw expression data can be found in Table S1. To determine significance, one-way ANOVAs were performed on raw expression values for each gene individually with Bonferroni *post hoc* tests to determine the significance between groups. ANOVA and Bonferroni results can be found in Table S2. Genes marked in bold are those for which Epas1 knockdown significantly (*p* < 0.05) increased expression.

The premise of this study is that birth imposes changes in fibroblasts which negatively impact the efficacy of fibroblast to cardiomyocyte reprogramming. To test this hypothesis, cardiac fibroblasts were isolated from both neonatal and adult mice. Identically passaged neonatal and adult cardiac fibroblasts were then transfected with miR combo. As expected, neonatal cardiac fibroblasts transfected with miR combo showed robust reprogramming as evidenced by increased expression of various cardiomyocyte-specific (Fig. 1*A*). In contrast, miR combo failed to increase the expression of any cardiomyocyte-specific genes in adult cardiac fibroblasts (Fig. 1*A*). To understand why reprogramming efficacy was weaker in cultured adult cardiac fibroblasts, we utilized our previously published RNA-seq data to identify a number of possible candidates (22). Candidates were chosen on the basis that they were down-regulated in fibroblasts reprogramming to cardiomyocytes. These candidates were Prrx1, Cebpd, Epas1, Mmp1b, Mmp8, Prrx1, and Sox9. All seven candidates were significantly enriched in adult cardiac fibroblasts when compared to neonatal cardiac fibroblasts (Fig. 1*B*). To determine the effect of these proteins on fibroblast to cardiomyocyte reprogramming, knockdown experiments were performed. Knockdown was robust for each candidate (Fig. 1*C*). Cultured adult cardiac fibroblasts were transfected with miR combo and a siRNA targeting one of the potential candidates. Cardiomyocyte gene expression was subsequently analyzed by qPCR. As shown in Figure 1*D*, the knockdown of Cebpd, Mmp8, Prrx1, and Sox9 had no effect on any cardiomyocyte gene tested. In contrast, robust expression of cardiomyocyte genes was observed in cultured adult cardiac fibroblasts transfected with miR combo and the siRNAs targeting Mmp1b or Epas1 (Fig. 1*D*). Based on the number of cardiomyocyte genes induced, we focused on Epas1.

**Figure 2:**
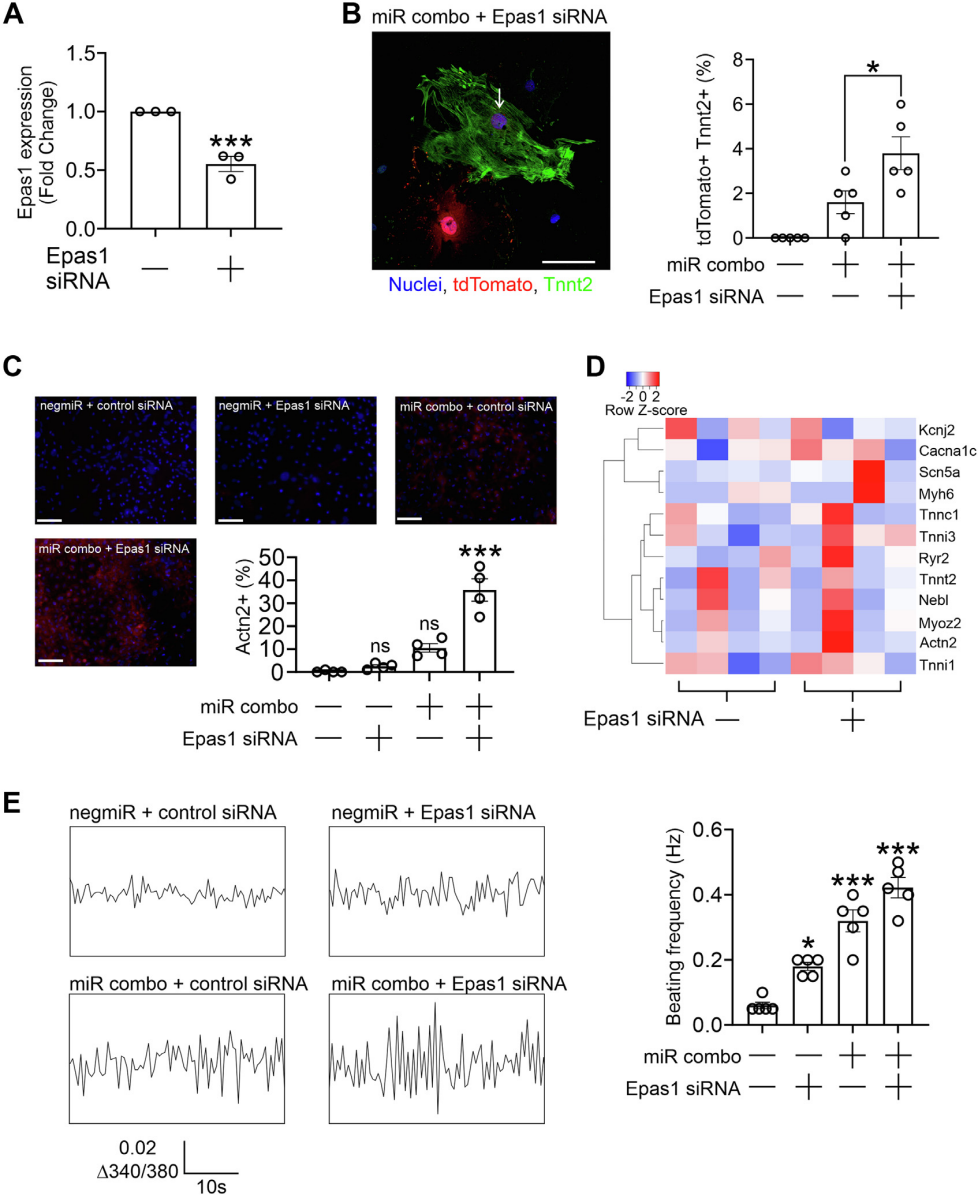
**Epas1 is expressed in adult cardiac fibroblasts and inhibits fibroblast to cardiomyocyte reprogramming.***A*, adult cardiac fibroblasts (1 passage after isolation) were transfected with either a control non-targeting siRNA or a siRNA targeting Epas1. After 3 days, RNA was extracted and analyzed for Epas1 mRNA levels by qPCR. Epas1 expression levels were normalized to the housekeeping gene Gapdh and are shown as a fold change. N = 3. ∗∗∗*p* < 0.001 (two-way independent *t* test). *B*, fibroblasts were isolated from hearts of adult fibroblast lineage tracing mice, Fsp1Cre:tdTomato. In these mice, fibroblasts express the fluorescent protein tdTomato. After one passage, cells were transfected with the indicated combination of miRNAs (negmiR or miR combo) and siRNAs (non-targeting control or a siRNA targeting one of the following: Prx1, Cebpd, Epas1, Sox9, Mmp1b, or Mmp8). After 14 days, cells were fixed and stained with antibodies targeting tdTomato (*red*) and the cardiomyocyte marker Tnni3 (*green*). Nuclei were counterstained with DAPI (*blue*). Representative images show cardiomyocytes derived from fibroblasts and quantification of the number of cardiomyocytes with observable sarcomeres provided. N = 5. ∗*p* < 0.05 (two-way independent *t* test between the indicated groups). Scale bar 100 micron. The *arrow* denotes a positive cell (TdTomato+ Tnni3+). *C*, cardiac fibroblasts (passage 1) derived from adult mice were transfected with siRNAs (control non-targeting or Epas1 targeting) and miRNAs (non-targeting control negmiR or the reprogramming cocktail miR combo). After 14 days, cells were fixed and stained with an Actn2 antibody (*red*). Nuclei were counterstained with DAPI (*blue*). Wide-field representative images are shown (scale bar 50 microns). The number of Actn2+ cells was counted and expressed as a percentage of the total number of cells. N = 4. One-way ANOVA, *F* (3, 12) = 38.34 *p* < 0.0001, with Bonferroni *post hoc* testing was used to determine the significance between groups. Significance to the control group (negmiR + control siRNA) is shown: ns-not significant, ∗∗∗*p* < 0.001. *D*, cardiac fibroblasts (passage 1) derived from adult mice were transfected with either a control non-targeting siRNA or a siRNA targeting Epas1. After 14 days, the expression of the indicated genes was determined by qPCR. Expression values were normalized to the housekeeping gene Gapdh. The heatmap shows individual expression values (as Z-scores) for three independent experiments. A two-way independent *t* test was used on the dataset and found no significant difference between the two groups for any gene tested (*p* > 0.05). *E*, cardiac fibroblasts (passage 1) derived from adult mice were transfected with siRNAs (control non-targeting or Epas1 targeting) and miRNAs (non-targeting control negmiR or the reprogramming cocktail miR combo). After 14 days, spontaneous calcium oscillations were determined. Representative traces are shown. The number of oscillations per second (Hz) was calculated from five individual traces. One-way ANOVA, *F* (3, 16) = 42.53 *p* < 0.0001, and Bonferroni *post hoc* tests were used to determine significance between the groups. Significances to the control group (negmiR + control siRNA) are shown: ∗*p* < 0.05, ∗∗∗*p* < 0.001.

Having demonstrated effects on cardiomyocyte gene expression, we then wanted to determine if Epas1 knockdown similarly enhanced miR combo efficacy with respect to cardiomyocyte generation. Epas1 knockdown in adult cardiac fibroblasts was robust (Fig. 2*A*). Two cardiomyocyte markers were used, Tnnt2 and Actn2. Irrespective of the marker used, the combination of miR combo and an Epas1 targeting siRNA increased the number of reprogramming events in cultured adult cardiac fibroblasts (Fig. 2, *B* and *C*). Epas1 knockdown alone had no effect on cardiomyocyte gene expression (Fig. 2*D*). Calcium oscillations increased in amplitude in response to Epas1 siRNA or miR combo transfection. Amplitude was greatest when Epas1 siRNA and miR combo were combined (Fig. 2*E*). While calcium oscillations were noted they were relatively small and lacked the typical rhythm when compared to cardiomyocytes derived from neonatal cardiac fibroblasts (2).

**Figure 3:**
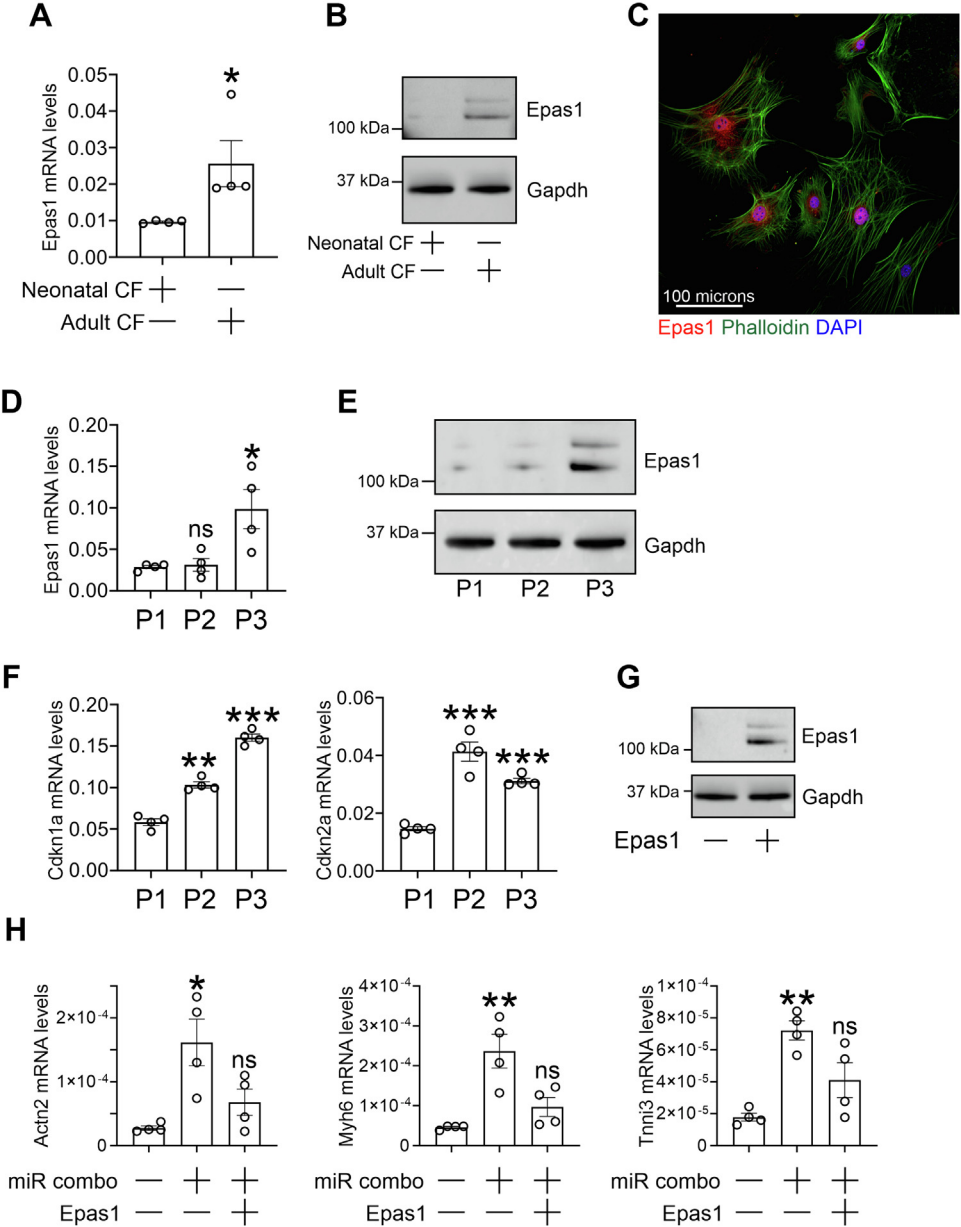
**Epas1 is a marker of fibroblast aging.***A*, fibroblasts were isolated from neonatal and adult cardiac tissue. After one passage, RNA was extracted and analyzed for the expression of Epas1 by qPCR. Epas1 expression values were normalized to the housekeeping gene Gapdh. N = 4. ∗*p* < 0.05 (two-way independent *t* test). *B*, fibroblasts were isolated from neonatal and adult cardiac tissue. After one passage, protein was extracted and analyzed for the expression of Epas1 by immunoblotting. Gapdh was used as a loading control. N = 4. Representative immunoblots are shown. *C*, fibroblasts were isolated from adult cardiac tissue. After one passage, cells were fixed and stained with an antibody targeting Epas1 (*red*). Cells were counterstained with phalloidin to mark the cytoskeleton (*green*) and DAPI to mark nuclei (*blue*). Representative image shown from three experiments. *D*, fibroblasts were isolated from neonatal cardiac tissue. RNA was isolated after one, two or three passages. Following extraction, RNA was analyzed for the expression of Epas1 by qPCR. Epas1 expression values were normalized to the housekeeping gene Gapdh. N = 4. One-way ANOVA, *F* (2, 9) = 8.897 *p* = 0.0074, with Bonferroni *post hoc* tests were used to determine significance. Comparisons are shown to the P1 group: ns-not significant, ∗*p* < 0.05. *E*, protein was also isolated after one, two, or three passages of neonatal cardiac fibroblasts and analyzed for Epas1 protein levels. Gapdh was used as the loading control. A representative image is shown from three separate experiments. *F*, fibroblasts were isolated from neonatal cardiac tissue. RNA was isolated after one, two, or three passages. Following extraction, RNA was analyzed for the expression of the cellular aging markers Cdkn1a and Cdkn2a by qPCR. Expression values were normalized to the housekeeping gene Gapdh. N = 4. One-way ANOVA, Cdkn1a: *F* (2, 9) = 81.97 *p* < 0.0001; Cdkn2a: *F* (2, 9) = 43.51 *p* < 0.0001, with Bonferroni post-hoc tests were used to determine significance. Comparisons are shown to the P1 group: ∗∗*p* < 0.01, ∗∗∗*p* < 0.001. *G*, fibroblasts were isolated from neonatal cardiac tissue. After one passage, cells were transfected with miRNAs (negmiR or miR combo) and plasmid DNA (empty plasmid or a plasmid containing an Epas1 expression cassette). Protein was extracted 3 days after transfection and immunoblotted. Immunoblots were probed with antibodies targeting Epas1 and Gapdh (loading control). Representative image shown from three independent experiments. *H*, fibroblasts were isolated from neonatal cardiac tissue. After one passage, cells were transfected with miRNAs (negmiR or miR combo) and plasmid DNA (empty plasmid or a plasmid containing an Epas1 expression cassette). After 14 days, expression of the indicated cardiomyocyte-specific markers was determined by qPCR. Expression values were normalized to the housekeeping gene Gapdh. N = 4. One-way ANOVA ,Actn2: F (2, 9) = 8.015 *p* = 0.0100; Myh6: *F* (2, 9) = 12.46 *p* = 0.0026, Tnni3: *F* (2, 9) = 13.79 *p* = 0.0018, with Bonferroni post-hoc tests were used to determine significance. Comparisons are shown to the control group: ns-not significant, ∗*p* < 0.05, ∗∗*p* < 0.01.

Epas1 protein was found to be weakly expressed in cultured neonatal cardiac fibroblasts and abundantly expressed in cultured adult cardiac fibroblasts (Fig. 3, *A* and *B*). In cultured adult cardiac fibroblasts, Epas1 was localized predominantly in the nucleus (Fig. 3*C*). Serial passaging of cultured neonatal cardiac fibroblasts was used to age the cells and thus mimic the transition of neonatal fibroblasts to adult fibroblasts. Serial passaging increased Epas1 mRNA (Fig. 3*D*) and Epas1 protein (Fig. 3*E*). This was concomitant with increased expression of the cell-cycle inhibitors Cdkn1a and Cdkn2a (Fig. 3*F*). To verify the negative impact of Epas1 on fibroblast to cardiomyocyte reprogramming, Epas1 was over-expressed in cultured neonatal cardiac fibroblasts (Fig. 3*G*). Epas1 over-expression was found to strongly inhibit reprogramming in cultured neonatal cardiac fibroblasts as evidenced by reduced expression of cardiomyocyte-specific genes (Fig. 3*H*).

**Figure 4:**
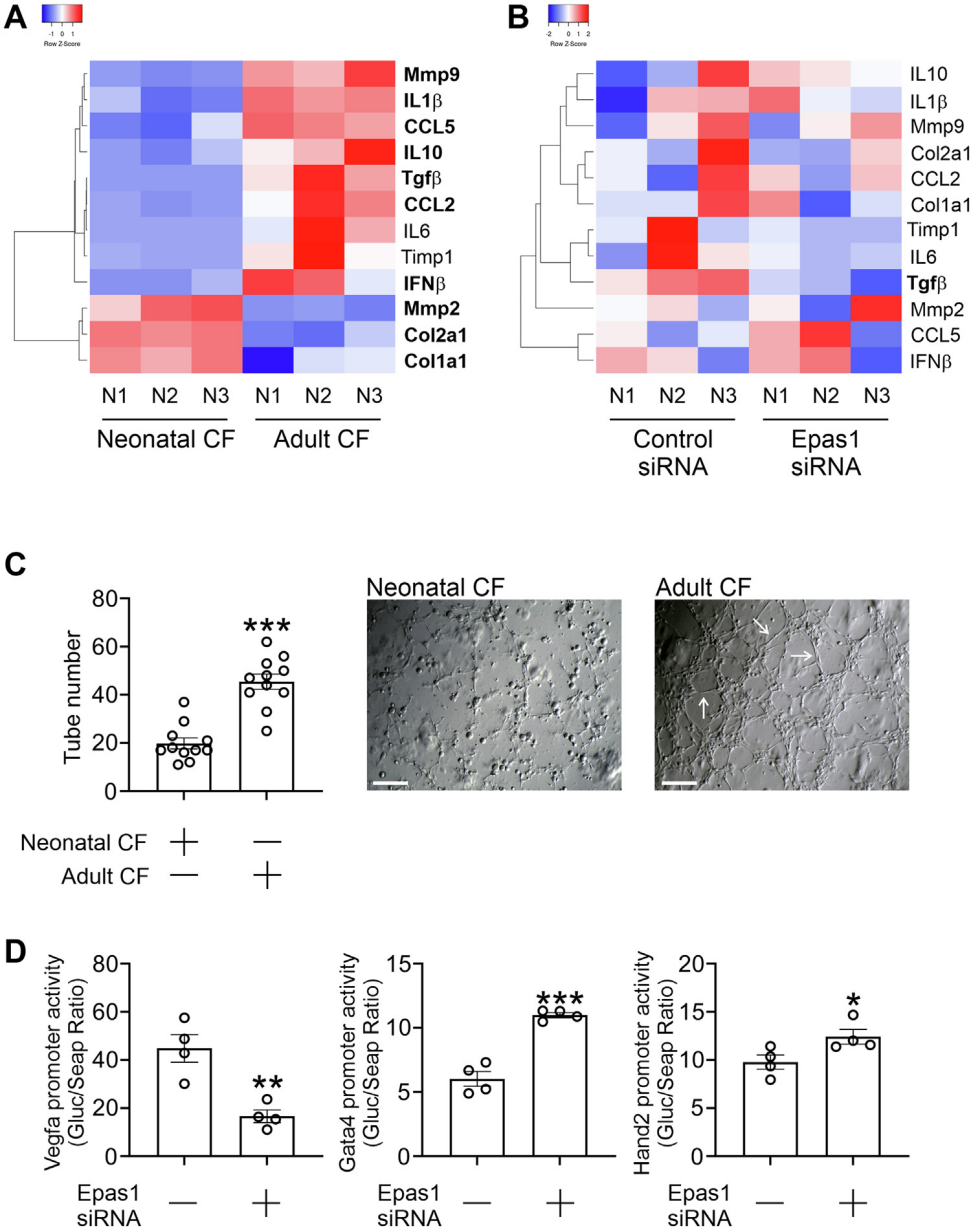
**Epas1 promotes fibroblasts to develop an angiogenic phenotype.***A*, neonatal and adult cardiac fibroblasts (passage 1) were analyzed for the expression of the indicated genes by qPCR. Expression values were normalized to the housekeeping gene Gapdh. The heatmap shows the individual expression values (as Z-scores) for three independent experiments. A two-way independent *t* test was used to determine significance between groups. Genes highlighted in bold were significantly different (*p* < 0.05) between neonatal and adult cardiac fibroblasts. Raw expression data can be found in Table S1. *B*, adult cardiac fibroblasts (passage 1) were transfected with either a control non-targeting siRNA or a siRNA targeting Epas1. After 3 days, the cells were analyzed for the expression of the indicated genes by qPCR. Expression values were normalized to the housekeeping gene Gapdh. The heatmap shows the individual expression values (as Z-scores) for three independent experiments. A two-way independent *t* test was used to determine significance between groups. Genes highlighted in bold were significantly different (*p* < 0.05) between the control and Epas1 siRNA groups. Raw expression data can be found in Table S1. *C*, fibroblasts were isolated from neonatal and adult cardiac tissue. After one passage, the cells were cultured under defined conditions to promote tube formation (angiogenesis). After 3 days, tube formation was visualized. Representative images are shown from 11 independent experiments. For each experiment, three fields were imaged and the tube number averaged. Scale bar 100 microns. The surface area for each imaged field was 0.40 mm^2^. Arrows denote tubes. A two-way independent *t* test was used to determine the significance between the two groups (∗∗∗*p* < 0.001). *D*, fibroblasts were isolated from adult cardiac tissue. After one passage, the cells were transfected with a siRNA (non-targeting control, Epas1 targeting) and two plasmids (Gluc and Seap). The Gluc plasmid contains a promoter (Vegf, Gata4, or Hand2) coupled to firefly luciferase. The Seap plasmid contains renilla luciferase and is used to normalize for transfection efficiency. Three days after transfection, luciferase measurements were made and are expressed as the Gluc/Seap ratio. N = 4. ∗*p* < 0.05, ∗∗*p* < 0.01, ∗∗∗*p* < 0.001 (two-way independent *t* test).

To understand how Epas1 was affecting fibroblast to cardiomyocyte reprogramming, we first analyzed the transition of neonatal to adult cardiac fibroblasts with respect to inflammation and extracellular matrix (ECM) production. Generally, the transition of neonatal to adult cardiac fibroblasts was associated with increased expression of inflammatory proteins (Fig. 4*A*). The effects on ECM proteins were more mixed, and unexpectedly fibroblast markers such as Col1a1 were found to decrease (Fig. 4*A*). Of all the tested inflammatory and ECM proteins, Epas1 knockdown only influenced tubular growth factor β (TGFβ) expression (Fig. 4*B*). TGFβ plays many roles, of which one is promoting angiogenesis. Moreover, Epas1 is a transcription factor that is known to regulate vascular endothelial growth factor (VEGF) expression and appears to be necessary for the development of blood vessels. Thus, our data suggested that increased Epas1 expression in adult fibroblasts following birth may promote fibroblasts to acquire an angiogenic phenotype. To test this, neonatal and adult cardiac fibroblasts were cultured under conditions that promote angiogenesis. Neonatal cardiac fibroblasts failed to display any angiogenesis as evidenced by a failure to undergo tube formation (Fig. 4*C*). In contrast, cultured adult cardiac fibroblasts showed robust angiogenesis (Fig. 4*C*). Promoter activity assays confirmed that Epas1 was functioning as a transcription factor. In cultured adult cardiac fibroblasts, Epas1 knockdown decreased the activity of the VEGF promoter (Fig. 4*D*). Further confirming the role of Epas1 as an inhibitor of the cardiomyocyte lineage, Gata4 and Hand2 promoter activities were increased in Epas1 knockdown cells (Fig. 4*D*).

**Figure 5:**
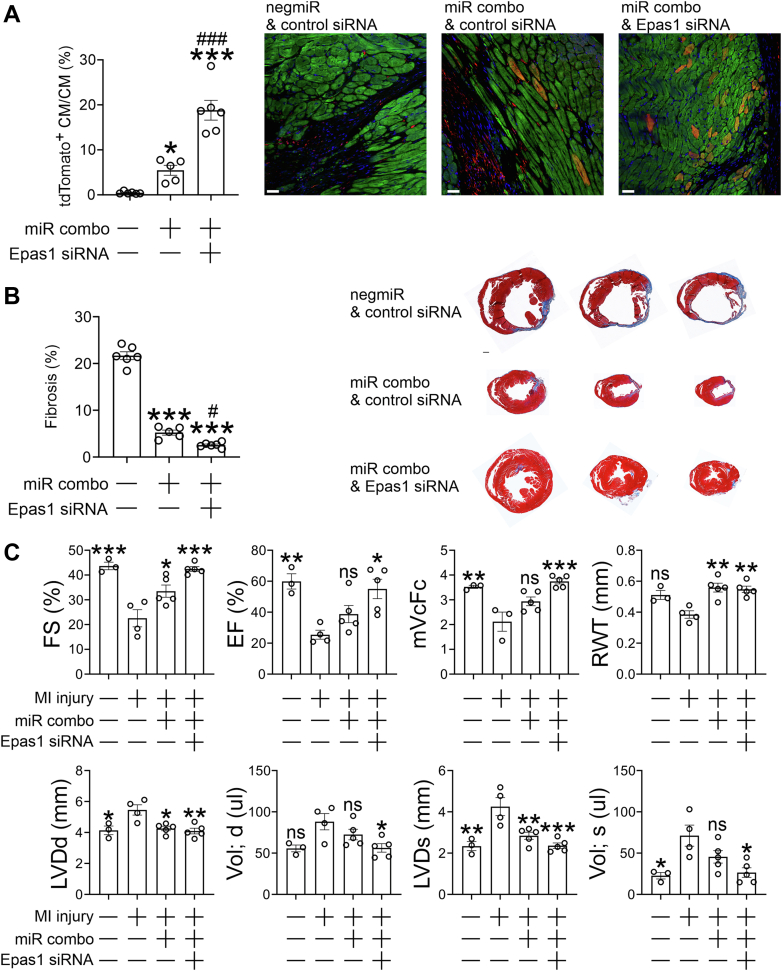
**Epas1 knockdown improves the efficacy of fibroblast to cardiomyocyte reprogramming *in vivo*.** Fibroblast lineage tracing Fsp1Cre:tdTomato mice were subjected to either a sham operation or myocardial infarction. Immediately after injury, fibroblast-targeting exosomes containing a combination of miRNAs and siRNAs were injected into the border zone. Mice received one of three combinations: (1) negmiR plus a non-targeting control siRNA; (2) miR combo plus a non-targeting control siRNA; or (3) miR combo plus an Epas1 targeting siRNA. *A*, reprogramming events 2 months post-injury. Cardiac tissue sections were incubated with antibodies targeting tdTomato or Tnni3 (cardiomyocyte marker). Sections were also counterstained with DAPI to visualize nuclei. Representative images from 5 to 6 mice per group are shown. The number of cardiomyocytes derived from fibroblasts (tdTomato+ Tnni3+) is expressed as a percentage of the total cardiomyocyte population (Tnni3+). N = 5 to 6 per group. One-way ANOVA, *F* (2, 14) = 44.22 *p* < 0.0001, and Bonferroni *post hoc* tests were used to determine significance. ∗Comparisons made to the negmiR + control siRNA group: ∗*p* < 0.05, ∗∗∗*p* < 0.001. #Comparisons made between the two miR combo groups: ###*p* < 0.001. Scale bar 50 microns. *B*, two months post-injury, cardiac tissue sections were stained with Masson’s Trichrome to quantify fibrosis. Representative serial sections are shown for each group. To determine the amount of fibrosis, the percentage of fibrosis over five serial sections was calculated and averaged. N = 5 to 6 per group. One-way ANOVA, *F* (2, 14) = 316.4 *p* < 0.0001, and Bonferroni post hoc tests were used to determine significance. ∗Comparisons made to the negmiR + control siRNA group: ∗∗∗*p* < 0.001. #Comparisons made between the two miR combo groups: #*p* < 0.05. Representative serial sections are shown. Scale bar 500 microns. *C*, cardiac function was determined 2 months post-injury. Functional parameters measured: FS (Fractional Shortening), EF (Ejection Fraction), mVcFc (mean velocity of circumferential fiber shortening), LVDd (Left ventricle diastolic diameter), Vol;d (Left ventricle volume in diastole), LVDs (Left ventricle systolic diameter), and Vol;s (Left ventricle volume in systole). One-way ANOVA and Bonferroni *post hoc* tests used to determine significance. Results of statistical testing can be found in Table S2. N = 3 to 6 per group. Significance to the control group (MI, negmiR + control siRNA) is shown: ns-not significant, ∗*p* < 0.05, ∗∗*p* < 0.01, ∗∗∗*p* < 0.001.

The data provided earlier suggested that Epas1 knockdown would improve the efficacy of fibroblast to cardiomyocyte reprogramming in adult mice. To test this, fibroblast lineage tracing mice were subjected to myocardial infarction. Following injury, miR combo and an Epas1 targeting siRNA were delivered into the infarct border zone. Two months after injury, the number of reprogramming events (tdTomato+ cardiomyocytes) was counted. In mice receiving both the non-targeting miRNA (negmiR) and a non-targeting siRNA, there were no tdTomato+ cardiomyocytes (Fig. 5*A*). As expected, the delivery of miR combo and a non-targeting siRNA induced reprogramming such that ∼5% of cardiomyocytes were derived from fibroblasts (Fig. 5*A*). Reprogramming events were increased even further in mice that had received both miR combo and the Epas1-targeting siRNA (Fig. 5*A*). Further studies were then conducted to determine the effects of Epas1 targeting on cardiac function and fibrosis. In agreement with our previous studies, miR combo reduced fibrosis significantly (Fig. 5*B*). Fibrosis was reduced further when an Epas1 targeting siRNA was added to miR combo (Fig. 5*B*). Similarly, as expected, miR combo also improved cardiac function (Fig. 5*C*). The improvements in cardiac function were enhanced by the addition of the Epas1 targeting siRNA (Fig. 5*C*). This appeared to be due to improved contractile behavior as noted by the improved systolic measurements and mean velocity of circumferential fiber shortening (Fig. 5*C*).

## Discussion

In this study, we have discovered that following birth, fibroblasts express Epas1 which promotes an angiogenic phenotype at the expense of reprogramming capacity. Reversing the phenotypic changes imposed by birth improved the efficacy of fibroblast to cardiomyocyte reprogramming.

*In situ* reprogramming of fibroblasts to cardiomyocytes has attracted much interest as a novel tool for cardiac regeneration (2, 3, 4, 5, 6, 7, 8, 9, 10, 11, 12). While various studies have demonstrated efficacy, the improvements in cardiac function are relatively modest (3, 12). Many researchers, including ourselves, have sought to improve efficacy through the identification and application of novel mechanistic insights. Such studies have identified potentially important roles for epigenetics and the innate immune system (5, 6, 14, 15, 16, 17). We, and others, identified that during fibroblast to cardiomyocyte reprogramming there is a significant loss of the epigenetic motif H3K27me3 (5, 14). Work in other laboratories suggests that the loss of H3K27me3 is part of a re-patterning of the epigenetic landscape with cardiomyocyte genes acquiring epigenetic signatures associated with gene activation such as H3K4me4 (14). However, it remains to be determined if such epigenetic changes are needed for reprogramming or are a consequence of reprogramming. Moreover, unless pharmacologic agents can be identified to target cardiomyocyte genes specifically, it is unclear how targeting generic epigenetic processes could be used clinically without inducing off-target events such as cancer. More recently, we discovered a novel role for pattern recognition receptors involved in regulating the innate immune response. Based on our studies, it appears that reprogramming factors weakly induce the pattern recognition receptor TLR3 (6). Enhancing TLR3 activation was found to enhance fibroblast to cardiomyocyte reprogramming efficacy *in vitro* (6). Similarly, single-cell studies by the Li group demonstrated that transcription factor–based methods of fibroblast to cardiomyocyte reprogramming also depend on TLR3 activation (23). The mechanistic studies described earlier have been important for furthering our understanding of fibroblast to cardiomyocyte reprogramming. Due to the apparent inability of adult cardiac fibroblasts to reprogram in culture, it has been necessary to conduct mechanistic studies solely with neonatal cardiac fibroblasts (5, 6, 14, 15, 16, 17). The potential issue is that findings from neonatal cardiac fibroblasts may not necessarily apply to adult cardiac fibroblasts. Indeed, neonatal and adult cardiac fibroblasts appear to be different from each other (18, 19, 20). Neonatal fibroblasts tend to produce more collagen, nestin, and smooth muscle actin than their adult counterparts (18, 19, 20). In contrast, higher expression of inflammatory cytokines is observed in adult fibroblasts (18, 19, 20). These changes appear to correlate with their regenerative capacity, with neonatal fibroblasts being associated with improved healing outcomes. In the tendon, for example, neonatal fibroblasts promote regeneration without scar formation while adult fibroblasts promote scar formation and suboptimal healing (20). In addition, cardiac fibroblasts appear to mimic cardiomyocytes in that their proliferation rate drops significantly after birth (19, 21). Cell cycle exit in cardiomyocytes following birth leads to cardiomyocyte maturation. By analogy, fibroblasts exiting the cell cycle after birth may also lead to fibroblast maturation (19, 21). We found that the transition of fibroblasts from a neonatal to adult phenotype negatively impacted reprogramming efficacy. Inducing cell cycle exit in neonatal cardiac fibroblasts by serial passaging, thereby mimicking the transition to adult fibroblasts, was found to inhibit fibroblast to cardiomyocyte reprogramming. Concomitant with cell cycle exit, fibroblasts began to express the transcription factor Epas1. Based on our data, Epas1 appears to be necessary for the differences in reprogramming efficacy between cultured neonatal and adult cardiac fibroblasts. This is based on two experiments. First, Epas1 knockdown in adult cardiac fibroblasts enabled reprogramming to cardiomyocytes. Second, Epas1 over-expression in neonatal cardiac fibroblasts prevented their reprogramming to cardiomyocytes. In essence, the data showed that manipulating the transition from the neonatal to adult fibroblast phenotype *in vitro* was a potentially viable strategy for enhancing reprogramming efficacy *in vivo*. Indeed, the knockdown of Epas1 *in vivo* was found to enhance the therapeutic benefits of fibroblast to cardiomyocyte reprogramming.

Known roles for Epas1 include promoting angiogenesis (24, 25, 26). Indeed, adult cardiac fibroblasts appeared to be primed to undergo angiogenesis as the cells were capable of differentiating into tube-like structures. In neonatal cardiac fibroblasts, where there is no Epas1 expression, there was no tube-like formation. While we observed that Epas1 activated angiogenic genes such as VEGFa, we also discovered that Epas1 inhibits the expression of cardiomyogenic genes such as Gata4. Context-dependent effects on transcription, whereby a transcription factor shows opposing effects on different groupings of genes, have been reported. For example, the transcription factor YY1 has been reported to both activate and repress gene transcription (27, 28, 29). While the mechanisms for context-dependent transcription appear complex, we are currently exploring the possibility that Epas1 functions as a repressor or an activator depending on the proteins already present in the gene. Recent work suggests that Epas1 may not be alone in influencing fibroblast cell fate. ChIP-seq and RNA-seq studies indicated that Mef2C, one of the constituents of the transcription factor cocktail for fibroblast to cardiomyocyte reprogramming, shifted ASCL1 binding from neuronal genes to cardiomyocyte genes (30). Another question is the signal which induces Epas1 expression. Based on our data, the signal is likely to be birth. Indeed, low oxygen levels, as found during birth, are a known stimulus of Epas1 expression (25). In this context, it is interesting to note that the low oxygen levels associated with birth have also been proposed as the stimulus driving cardiomyocyte cell cycle exit. We are also interested to identify why the effects of Epas1 knockdown *in vivo* were predominantly on systolic function. This may be related to the increased numbers of new cardiomyocytes in the infarct region, enabling enhanced contractility in the cardiac tissue.

Exosomes were used as the delivery agents in this study. Commonly, viruses are used as delivery agents. However, they are problematic in that certain viruses (retrovirus, lentivirus) are integrating and for others (AAVs) there exists a significant number of individuals with immunity against them. Moreover, viruses are difficult to adapt for multiplexing when various DNA/RNA molecules have to be expressed (31). An alternative is an exosome. In contrast to viruses, they are not recognized by the host immune system and they are naturally adapted to delivering multiple molecules. We have found that C166-derived exosomes are an effective delivery agent for multiple miRNAs (http://ssrn.com/abstract=4279247). Moreover, they provided an additional benefit in that they appear to be selective for cardiac fibroblasts *in vivo*. Using tracing molecules, we found that C166-derived exosomes were readily taken up by cardiac fibroblasts while rarely internalized by cardiomyocytes or endothelial cells (http://ssrn.com/abstract=4279247). The current study is important in that it demonstrates that C166-derived exosomes can effectively deliver miRNAs and siRNAs.

## Experimental procedures

### Mouse cardiac fibroblasts

Cardiac fibroblasts were derived from 1-day-old neonate C57BL6 and 8-week-old mice and cultured according to established protocols (4).

### Exosome isolation

C166 cells were seeded with exosome-free serum in T75 flasks at a concentration of 1 × 10^6^ cells per flask. Forty-eight hours later, the supernatant was collected, centrifuged at 1000*g* for 10 min at 4 °C to remove non-adherent cells, and filtered through a 0.22-μm filter. The filtrate was then ultracentrifuged at 120,000*g* for 70 min at 4 °C. The exosome pellet was re-suspended in PBS and ready for use.

### Lipid-based transfection *in vitro*

Following isolation, fibroblasts were cultured in growth media containing DMEM (ATCC, Catalogue number 30-2002) supplemented with 15% v/v FBS (Thermo Scientific Hyclone Fetal bovine serum, Catalogue number SH30071.03, Lot number AXK49952) and 1% v/v penicillin/streptomycin (Gibco, Catalogue number 15140-122, 100 units Penicillin, 100 μg/ml Streptomycin). Fibroblasts were passaged once the cells had reached 70 to 80% confluence using 0.05% w/v trypsin (Gibco, Catalogue number 25300-054). After one passage, the cells were used for transfection. For all experiments, cells were seeded at 5000 cells/cm^2^ in growth media. After 24 h, the cells were transfected with 5 nmol miRNA (negmiR or miR combo: ThermoScientific) and/or 5 nmol siRNA (control or targeting: Dharmacon) *via* the lipid-based transfection reagent Dharmafect-I (ThermoScientific) according to manufacturer’s instructions. Transfection complexes were removed after 24 h, and cells were cultured in growth media for the duration of the experiment.

### qPCR

Total RNA was extracted using Quick-RNA MiniPrep Kit according to the manufacturer’s instructions (Zymo Research). Total RNA (50 ng–100 ng) was converted to cDNA using a high-capacity cDNA reverse transcription kit (Applied Biosystems). cDNA was used in a standard qPCR reaction involving FAM conjugated gene-specific primers (ThermoFisher) and TaqMan Gene Expression Master Mix (ThermoFisher). Primers were acquired from ThermoFisher (2, 5, 6).

### Immunoblotting

The cell layer was washed once with PBS and proteins lysed with 150 μl lysis buffer (62.5 mM Tris pH 8, 1% v/v SDS, 1% v/v mammalian protease inhibitor cocktail (Sigma), 10% v/v phosphatase inhibitor cocktail (Roche, stock one tablet in 1 ml water) per well of a 6-well plate on ice. The Epas1 antibody (Novus Biological Cat no NB100-122) and HRP-conjugated secondary antibodies (Cell Signaling) were used according to the manufacturer’s instructions. Proteins were visualized by chemiluminescence using ECL-Plus (GE Healthcare), and bands were visualized and quantified using a Syngene G-box and attendant software.

### Immunostaining of cultured cells

Cells were fixed with 2% v/v paraformaldehyde (EMS) as described previously (32). Fixed cells were blocked in antibody buffer (5% w/v BSA, 0.1% v/v Tween-20, in PBS) for 1 h at room temperature. Following blocking, cells were incubated overnight at 4 °C with a targeting antibody (Epas1: Novus Biological Cat no NB100-122; Tnnt2: Abcam Cat no ab45932) in antibody buffer. After the overnight incubation, cells were washed three times in an antibody buffer. Following washing, cells were incubated with Alexa-Fluor conjugated secondary antibodies (ThermoFisher) at a 1:500 dilution in antibody buffer for 1 h at room temperature. Nuclei were stained by DAPI at 1 μg/ml for 30 min at room temperature in antibody buffer. Phalloidin was used per manufacturers’ instructions (ThermoFisher). Following washing in PBS to remove unbound complexes, immunofluorescence was measured using a Zeiss Axiovert 200 inverted microscope.

### Calcium imaging and contractility

Calcium signals in cultured cardiac fibroblasts were imaged using Fura-2 according to previously published protocols (33). Fura-2 AM loading (1 μM; Molecular Probes/Invitrogen) and calcium imaging were performed in Ringer solution (140 mM NaCl, 2.8 mM KCl, 2 mM CaCl2, 2 mM MgCl2, 10 mM glucose, 10 mM HEPES, pH 7.4). Experiments were carried out at room temperature. To determine if cells exhibited spontaneous oscillations, >8 fields of cells in each dish were imaged for at least 3 min each. Once oscillations were observed, cells were imaged for at least 5 min, and further fields were tested until no additional oscillating cells were found.

### Myocardial infarction and delivery of miRNAs and siRNAs

Adult male fibroblast-specific protein 1 Cre-tandem dimer Tomato (Fsp1-Cre:tdTomato) mice were subjected to permanent ligation of the left anterior descending coronary artery as described previously (3). C166-derived exosomes (20 μl; from 1 × 10^6^ cells) loaded with 100 pmol total of miRNAs (non-targeting negmiR or miR combo) and siRNAs (non-targeting or targeting Epas1) and were injected at two sites 2 mm below the site of ligation. For the sham operation, the chest cavity was opened and closed.

### Immunocytochemistry

Hearts were removed and fixed in formalin. After sectioning, sections were stained with antibodies for cardiac troponin-T (Abcam) and tdTomato (Abcam). Confocal images were captured using an LSM 510 Meta DuoScan microscope (Zeiss) and processed using LSM five software, version 4.2.

### Fibrosis measurements

Hearts were removed and fixed in formalin. After sectioning, serial sections at 200-micron intervals through the infarct zone were stained with Masson’s Trichrome. Images were captured with Axio Imager upright microscope and data were processed with ImageJ. Fibrosis measurements are reported as the percentage area of the left ventricle.

### Echocardiography

B-mode and M-mode echocardiography was carried out by the Duke Cardiovascular Research Center Core Facility as reported previously.

### Images

Images were processed with CorelDraw and Zeiss software (Axiovision Rel4.8 and Zen Blue).

### Statistics

All statistical analysis was performed using GraphPad. T-Tests (two groups) and ANOVAs (>two groups) were used as appropriate. For ANOVA, Bonferroni *post hoc* tests were used to determine the significance between groups. Individual data points and a summary bar graph of Mean ± SEM is shown. A *p*-value of <0.05 was considered significant.

### Animal study approvals, anesthesia, and euthanasia

Experiments using animals were approved by the Duke University Division of Laboratory Animals and the Duke Institutional Animal Care and Use Committee. In addition, all studies and procedures conformed to the NIH Guide for the Care and Use of Laboratory Animals. Where necessary, anesthesia was carried out with a single i.p. injection of ketamine (100 mg/kg) and xylazine (5 mg/kg). Mice were euthanized *via* decapitation (neonatal mice) or *via* saturating atmosphere of isoflurane followed by cervical dislocation.

## Data availability

All of the data is contained within the manuscript and Supporting Tables.

## Supporting information

This article contains supporting information.

## Conflict of interest

Conrad P. Hodgkinson and Victor J. Dzau are co-founders of Recardia Therapeutics. This company aims to translate miR combo to clinical applications. The remaining authors have no conflicts of interest.
